# Stem cell–associated osteogenic deficiency causes craniofacial deformities with progeroid accumulation of prelamin A

**DOI:** 10.1172/jci.insight.196932

**Published:** 2026-02-03

**Authors:** Kai Li, Trunee Hsu, Hitoshi Uchida, Tingxi Wu, Susan Michaelis, Howard Worman, Wei Hsu

**Affiliations:** 1 The ADA Forsyth Institute, Somerville, Massachusetts, USA.; 2 Case Western Reserve University, Cleveland, Ohio, USA.; 3 The Johns Hopkins University School of Medicine, Baltimore, Maryland, USA.; 4 Vagelos College of Physicians and Surgeons, Columbia University, New York, New York, USA.; 5 Faculty of Medicine of Harvard University, Cambridge, Massachusetts, USA.; 6 Harvard School of Dental Medicine, Boston, Massachusetts, USA.; 7 Harvard Stem Cell Institute, Cambridge, Massachusetts, USA.

**Keywords:** Aging, Cell biology, Development, Bone development, Cytoskeleton, Stem Cells

## Abstract

Mutations in *LMNA*, encoding nuclear lamina protein Lamin A/C, cause premature aging disorders, most notably Hutchinson-Gilford progeria syndrome. Despite obvious skull abnormalities in patients with progeria, the etiology remains elusive. The L648R single–amino acid substitution blocks prelamin A maturation in mice, modeling a unique patient. Here, we identify prelamin A accumulation as a causative link to craniosynostosis in low bone density, contrasting conventional suture fusion in excessive ossification. The mutation causes skeletal stem cell deficiencies and subsequent osteogenesis. Intrasutural bones present in patients with progeria resemble synostosis caused by stem cell exhaustion. Comparative gene expression profiling further reveals cytoskeletal dynamics associated with skeletogenic cell aging and suture patency in mice and humans. Functional studies demonstrate that abnormal structures of progeric nuclei affect cytoskeleton organization and nucleoskeleton assembly essential for craniofacial skeletogenesis. Our findings provide compelling evidence for nuclear and cytoskeletal defects, causing stem cell–associated osteogenic defects in progeroid disorders.

## Abstract 2

Craniosynostosis in mice with progeroid Lmna mutation

## Graphical abstract

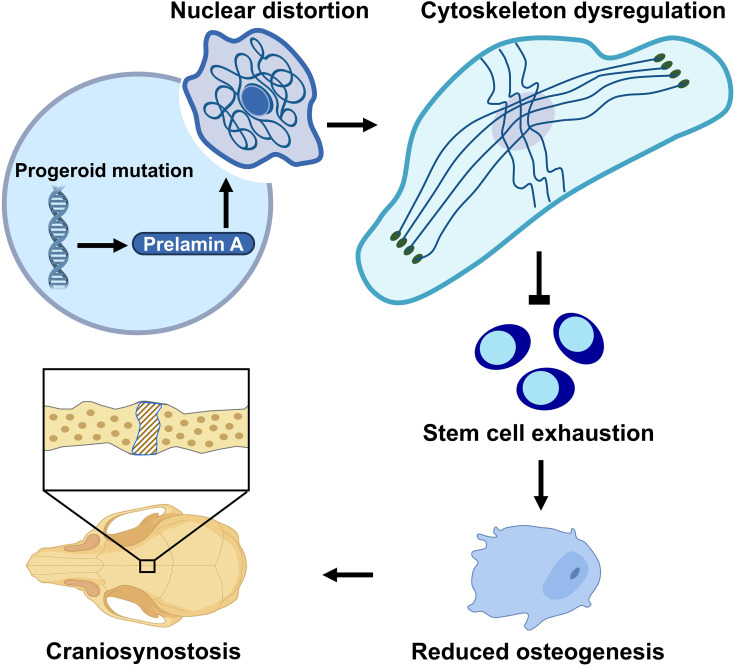

## Introduction

Mutations in *LMNA*, which encodes nuclear lamina proteins lamin A and lamin C, cause inherited diseases known as laminopathies. Among the best characterized of these is the premature-aging-like Hutchinson-Gilford progeria syndrome (HGPS) (1, 2). Prelamin A, the precursor of lamin A, has a carboxyl-terminal cysteine-aliphatic-aliphatic-any amino acid (CAAX) motif that promotes posttranslational processing to yield farnesylated and carboxyl methylated prelamin A (3–5). Almost immediately after its synthesis, the last 15 amino acids of prelamin A, including its farnesylated cysteine, are cleaved off by the zinc metalloprotease ZMPSTE24 to produce nonfarnesylated mature lamin A (6–8). When cleavage by ZMPSTE24 is blocked, farnesylated prelamin A accumulates, leading to progeroid diseases, with associated abnormalities in nuclear morphology (9, 10).

HGPS is caused by a splicing mutation in *LMNA,* generating an internally truncated variant of prelamin A called progerin (1, 2). Progerin retains its CAAX motif but lacks the ZMPSTE24 cleavage site, remaining farnesylated and methylated at the carboxyl terminus (11, 12). A related progeroid disorder, mandibuloacral dysplasia type B (MAD-B), results from mutations in *ZMPSTE24*, leading to the accumulation of full-length farnesylated prelamin A, and can have clinical presentations either more or less severe than HGPS (13, 14). We previously reported a unique patient with a phenotype similar to MAD-B but designated as MAD-B–like due to a genetic mutation in *LMNA* rather than *ZMPSTE24* (15). This patient has a heterozygous *LMNA* L647R point mutation that alters the leucine adjacent to the ZMPSTE24 cleavage site in human prelamin A, thereby resulting in full-length farnesylated prelamin A accumulation. While progerin (causes HGPS) and full-length prelamin A (causes MAD-B and MAD-B–like) are both drivers of disease, they appear to have overlapping but distinct cellular effects. Several studies have examined the effects of Progerin on bone (9, 16–19). Our characterizations of a mouse model (*Lmna^L648R^*) with the corresponding MAD-B–like prelamin A L648R amino acid substitution indicate osteoporotic defects caused by prelamin A accumulation (20). *Lmna^L648R^* mutant mice also exhibit mineralization defects in the skeleton, mandible, temporomandibular joint, and teeth (20). As a result, this animal model is valuable for gaining mechanistic insights into developmental and pathogenic processes directly attributed to the accumulation of full-length prelamin A. Because accumulation of prelamin A has been proposed, and in 1 report associated with physiological aging (21–23), a mechanistic understanding of prelamin A’s detrimental effects could be valuable for understanding molecular processes in bone maintenance that go awry in aging.

The abnormal skull appearance in patients with HGPS or MAD-B resembles that of craniosynostosis, a childhood disease caused by premature closure of cranial sutures (24). The suture, equivalent to the growth plate in the long bone, serves as a growth center regulating calvarial skeletogenesis and homeostasis (25). Skeletal stem cells (SSCs) residing in the suture mesenchyme possess unique properties and are naturally programmed for osteoblast differentiation to form intramembranous bones (25, 26). Calvarial bone formation is thus mediated by intramembranous ossification, a process not involving a cartilage intermediate and thus distinct from endochondral ossification in the body skeleton (27). Excessive intramembranous ossification has been well established to promote suture fusion (28), including the loss of AXIN2, resulting in craniosynostosis in mice and humans (29, 30). Synostosis can also be caused by cell fate switching, leading to suture closure via endochondral ossification (26). The expression of *Axin2* in the presumptive niche site is tightly linked to suture patency, which permits our purification and characterization of mouse and human suture stem cells (SuSCs) — SSCs within the suture mesenchyme (25, 31). This has led to our discovery of craniosynostosis caused by stem cell depletion (31). SuSCs that express AXIN2 exhibit long-term self-renewal, clonal expansion, multipotency, and skeletogenic potential and are genuine SSCs essential for calvarial bone development, homeostasis, and injury-induced repair (25, 32).

Despite the obvious skull abnormality exhibited in patients with HGPS and MAD-B, the mechanism underlying these deformities remains poorly understood. To elucidate the mechanism by which prelamin A impairs skull development, we use a mouse model homozygous for the *Lmna* mutant allele with L648R amino acid substitution (hereafter referred to as *Lmna^L648R^*), which alters the leucine residue adjacent to the ZMPSTE 24 cleavage site in prelamin A. The progeroid mutation of *Lmna^L648R^* blocks the proteolytic conversion of prelamin A to mature lamin A, resulting in the accumulation of permanently farnesylated prelamin A. The *Lmna^L648R^* mice exhibit multiple suture synostosis in low bone density, contrasting with conventional craniosynostosis in excessive ossification. These phenotypic defects are highly reminiscent of Wormian bones, which are intrasutural bones present in some craniosynostosis patients, as well as patients with progeroid MAD-B, implying a stem cell deficiency (33–35). Bioinformatic analysis of datasets from single-cell transcriptomics of aging mice and gene expression profiling of human nonsyndromic synostosis patients further helps decipher the disease culprit, tightly associated with the nucleus-cytoskeleton. Our findings have led to the unveiling of a functional connection between nuclear-cytoskeleton organization and craniofacial deformities caused by prelamin A accumulation in stem cell–associated osteogenic defects.

## Results

### Craniosynostosis in Lmna^L648R^ progeroid mice.

**Figure 1:**
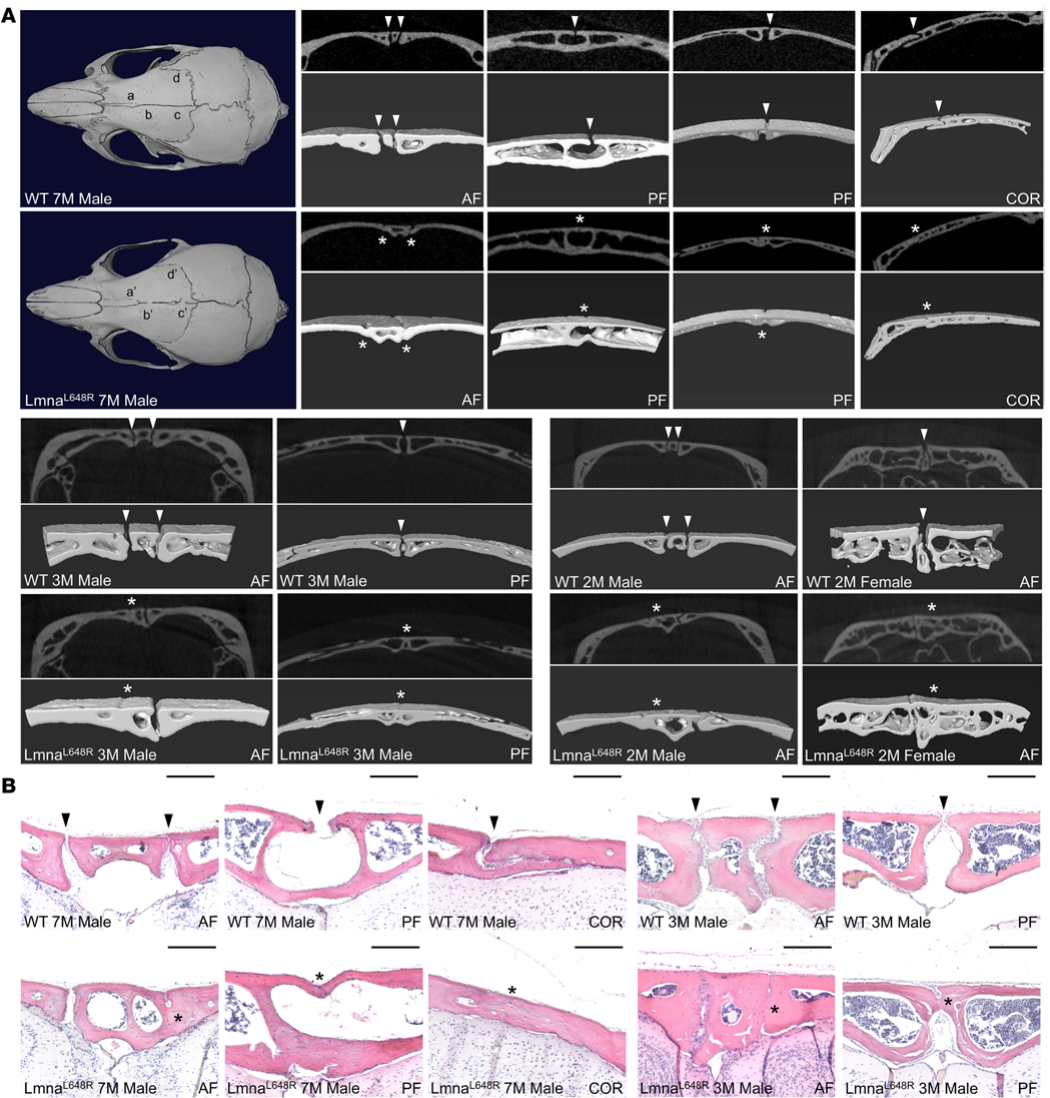
Multiple suture synostosis caused by *Lmna^L648R^* mutation. (**A**) Representative μCT-scanned coronal sections and 3D renderings of *Lmna^+/+^* (WT) and *Lmna^L648R/L648R^* (*Lmna^L648R^*) male and female skulls at 2, 3, and 7 months. (**B**) H&E staining of 3-month and 7-month-old WT and *Lmna^L648R^* calvarial sections. Arrowheads and asterisks indicate patent and synostosed sutures, respectively. AF, anterior frontal; COR, coronal; PF, posterior frontal. Scale bars: 200 μm.

Using the *Lmna^L648R^* mouse model (20), we further investigated the mechanistic cause of skull abnormalities associated with progeroid disorders. μCT analysis of WT and mutant mice revealed that the *Lmna^L648R^* mutation causes calvarial deformities with the closure of multiple sutures, including anterior frontal (AF), posterior frontal (PF), and coronal (COR) sutures (Figure 1A and Supplemental Figure 1; supplemental material available online with this article; https://doi.org/10.1172/jci.insight.196932DS1). Both 2D and 3D-rendered images suggest a unilateral or bilateral fusion of the AF suture, the closure of the ectocranial layer in the PF suture, and the disappearance of the COR suture (Figure 1A and Supplemental Figure 1). Craniosynostosis could be detected in the *Lmna^L648R^* mice as early as 2 months, with evidence showing effects on multiple sutures at 3 months (Figure 1A and Supplemental Figure 2). Multiple suture synostosis was then demonstrated by histological evaluation of H&E staining, which provides a definitive assessment of skull deformities (Figure 1B and Supplemental Figure 3). These data provide compelling evidence supporting that the accumulation of farnesylated prelamin A caused by disruption of ZMPSTE24 cleavage leads to aberrant suture closure and craniofacial bone abnormalities in progeroid diseases.

### Effects of Lmna^L648R^ mutation on stem cell–mediated osteogenesis.

**Figure 2:**
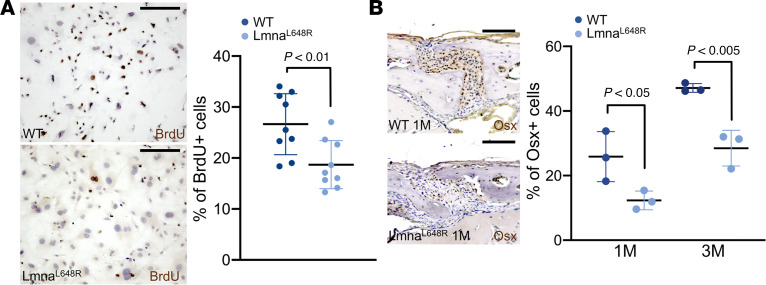
Expansion of osteoprogenitors affected by progeroid accumulation of prelamin A. (**A**) Representative images of BrdU staining examine the proliferation rate of calvarial cells isolated from *Lmna^+/+^* (WT) and *Lmna^L648R/L648R^* (*Lmna^L648R^*) mice. Graphs indicate quantitation of the average percentage of BrdU^+^ cells in 4 independent experiments (*P* < 0.01, *n* = 9, mean ± SD, 2-tailed Student’s *t* test). (**B**) Representative images of Osterix (Osx) staining analyze the osteoprogenitor cells within the 1-month-old (1M) WT and *Lmna^L648R^* cranial sutures. Graphs indicate the quantitation of the average percentage of Osx^+^ cells in 3 independent experiments (*P* < 0.05 or 0.005, *n* = 3, mean ± SD, 2-tailed Student’s *t* test). Scale bars: 200 μm (**A**) and 100 μm (**B**).

**Figure 3:**
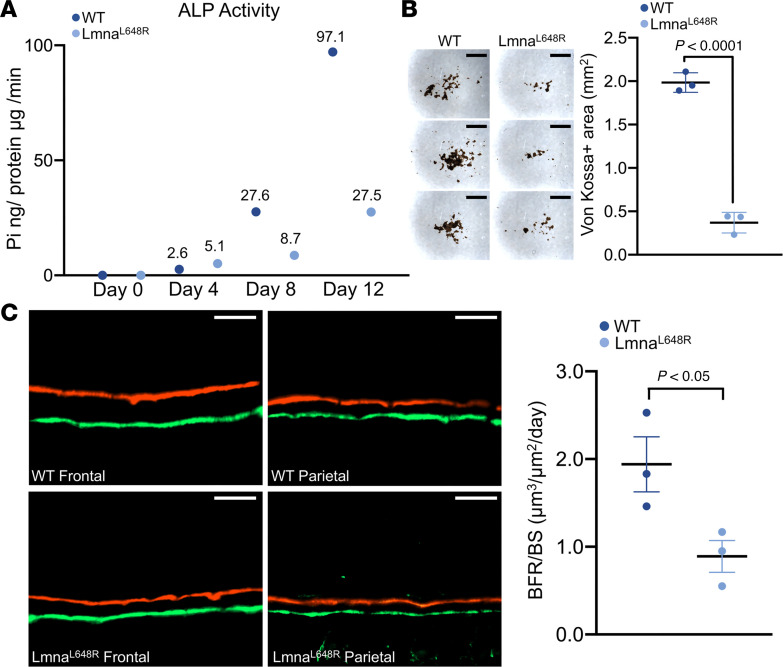
Progeroid accumulation of prelamin A impairs osteogenesis. (**A**) Graph is a representative of three independent experiments analyzing alkaline phosphatase (ALP) activity of WT and *Lmna^L648R^* calvarial cells cultured in differentiation media for days as indicated. (**B**) Representative images of von Kossa staining examine mineralized nodule formation in the culture of WT and *Lmna^L648R^* calvarial cells for 21 days. Graphs indicate the quantitation of the average stained area in 6 independent experiments (*P* < 0.0001, *n* = 3, mean ± SD, 2-tailed Student’s *t* test). (**C**) Alizarin red and calcein double labeling analysis examines mineralized tissue growth in frontal and parietal bones over 2 weeks. Graphs indicate the average bone formation rate of 2-month-old WT and *Lmna^L648R^* mice (*P* < 0.05, *n* = 3, mean ± SD, 2-tailed Student’s *t* test). Scale bars: 2 mm (**B**) and 50 μm (**C**).

To elucidate the mechanism underlying calvarial deformities caused by the *Lmna^L648R^* mutation, we first examined the proliferation and differentiation of osteoblast cells. Calvarial cells, which include osteogenic precursors, were isolated from the WT and *Lmna^L648R^* mutant calvaria at P4 and analyzed by transient BrdU labeling to examine cell proliferation. We found a significant reduction in the ex vivo expansion of mutant calvarial cells compared with the WT (Figure 2A and Supplemental Figure 4; *P* < 0.01, *n* = 9, mean ± SD, 2-tailed Student’s *t* test). Immunostaining of osterix (Osx) indicated that the number of osteoprogenitor cells is also significantly reduced in the suture of *Lmna^L648R^* mice (Figure 2B and Supplemental Figure 5; *P* values as indicated, *n* = 3, mean ± SD, 2-tailed Student’s *t* test). Both in vivo and ex vivo analyses thus suggested the *Lmna^L648R^* mutation has negative effects on the production of the osteogenic precursor population. Next, we examined osteoblast differentiation and mineralized nodule formation using alkaline phosphatase and von Kossa staining. It is not feasible to use SuSCs for these assays because a large number of committed osteogenic cells are required to generate detectable alkaline phosphatase–positive areas and von Kossa–positive mineralized nodules for 2–4 weeks in ex vivo differentiation. Therefore, calvarial osteogenic cells, which can be obtained in larger quantities, were used for these ex vivo analyses. The results indicate osteoblastogenesis is significantly repressed by the progeroid mutation (Figure 3, A and B, and Supplemental Figure 6; *P* < 0.0001, *n* = 3, mean ± SD, 2-tailed Student’s *t* test). Furthermore, dynamic measurement of mineral deposition by alizarin red and calcein double labeling analysis demonstrated a significant reduction in bone formation rate in the mutants (Figure 3C; *P* < 0.05, *n* = 3, mean ± SEM, 2-tailed Student’s *t* test).

**Figure 4:**
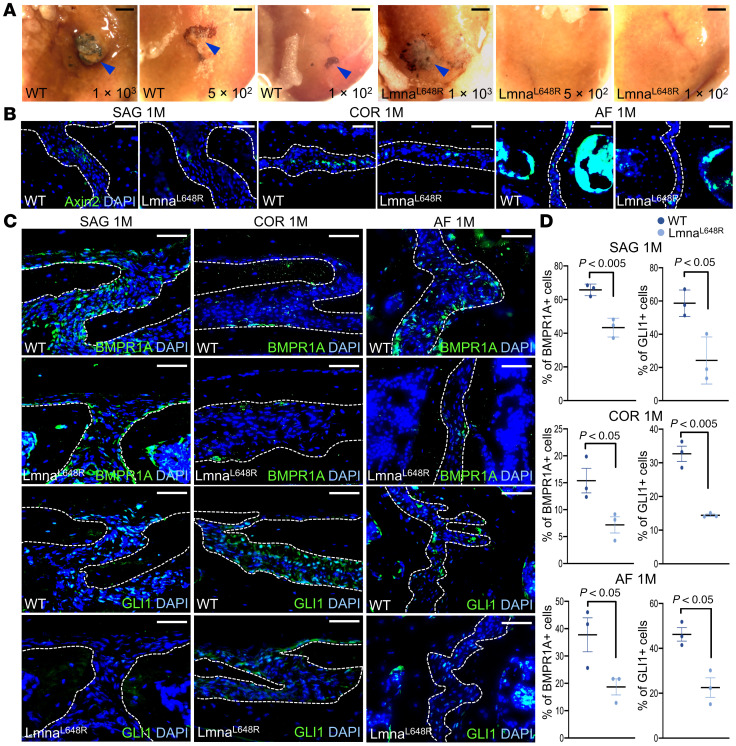
Suture stem cell deficiency in *Lmna^L648R^* mouse model for human progeroid disorder. (**A**) Limiting dilution analysis of stem cell–mediated bone formation with renal capsule transplantation. Representative images of whole-mount von Kossa staining detecting ectopic bone formation in the mouse recipients transplanted by the indicated number of suture cells into the renal capsule. Arrowheads indicate the ectopic bones. (**B**) Representative images showing the analysis of Axin2-expressing cells using the *Axin2^mGFP^* allele in the indicated 1-month-old (1M) suture. (**C**) Representative images examining the BMPR1A^+^ and GLI1^+^ cell population within the indicated 1-month-old (1M) suture. (**D**) Graphs indicate the quantitation of the average percentage of BMPR1A^+^ and GLI1^+^ cells in 3 independent experiments (*P* < 0.005 or 0.05, *n* = 3, mean ± SEM, 2-tailed Student’s *t* test). SAG, sagittal; COR, coronal; AF, anterior frontal. Scale bars: 1 mm (**A**) and 50 μm (**B** and **C**).

Craniosynostosis is mainly associated with excessive intramembranous ossification (24). However, the *Lmna^L648R^* mutants display suture synostosis in the setting of reduced osteogenic conditions, which is unusual. One possibility is apoptosis, previously implicated in suture closure (36), but there was no indication of cell death contributing to the synostosis phenotype (Supplemental Figure 7). Craniosynostosis, mediated by excessive ossification, generally affects a single suture. We hypothesize that multiple suture synostoses are linked to stem cell deficiency, as first described in SuSC-specific disruption of a BMP type 1 receptor, BMPR1A (31). The presence of SuSCs is essential for the maintenance of the stem cell niche and suture patency (31). To test our hypothesis on stem cell defects, we examined the characteristics of *Lmna^L648R^* SuSCs. By in vivo clonal expansion analysis, we previously showed the ability of a single Axin2^+^ SuSC to generate calvarial bone upon implantation into the kidney capsule (25). Limiting dilution analysis further provided a quantitative method to examine stem cell clonal expansion in the transplanted kidney, thereby measuring stem cell frequency (25). We used this rigorous assay to determine if the *Lmna^L648R^* mutation affects the clonal expansion and number of SuSCs. Varying amounts of cells isolated from the P7–P10 control and *Lmna^L648R^* sutures were implanted into the renal capsule, followed by the analysis of ectopic bone formation in the implanted site using von Kossa staining (Figure 4A). Transplantation of 5 *×* 10^2^ to 2.5 *×* 10^4^ WT suture cells had a 100% success rate of bone formation (Table 1). At 1 *×* 10^2^ cells from WT, ectopic bone was formed, although at a much lower rate (Table 1). In contrast, ectopic bone tissue was not always detected in kidneys transplanted with 5 *×* 10^2^ and 1 *×* 10^3^ *Lmna^L648R^* suture cells and was undetectable with 1 *×* 10^2^ *Lmna^L648R^* suture cells (Table 1). For WT at this stage, the estimated stem cell frequency is 1 in 208 suture cells, similar to our prior report (31). However, the *Lmna^L648R^* mutation significantly decreases SuSC frequency to 1 in 845 suture cells (Table 1; *P* = 0.0218). Moreover, examination of the stem cell marker showed a reduction of Axin2-expressing cells in the sagittal, COR, and AF suture (Figure 4B). Immunostaining of BMPR1A and GLI1 expressed in SuSCs showed their significant loss in the *Lmna^L648R^* suture (Figure 4, C and D, and Supplemental Figure 8; BMPR1A: SAG, WT, 65.76% ± 3.46%; *Lmna^L648R^*, 43.36% ± 5.62%, *P* < 0.005; COR, WT, 15.39% ± 2.28%; *Lmna^L648R^*, 7.18% ± 1.50%, *P* < 0.05; AF, WT, 37.76% ± 6.20%; *Lmna^L648R^*, 18.70% ± 2.92%, *P* < 0.05, *n* = 3, mean ± SEM, 2-tailed Student’s *t* test, GLI1: SAG, WT, 58.67% ± 4.59%; *Lmna^L648R^*, 24.20% ± 8.17%, *P* < 0.05, COR, WT, 32.69% ± 2.26%; *Lmna^L648R^*, 14.42% ± 0.35%, *P* < 0.005; AF, WT, 46.22% ± 3.02%; *Lmna^L648R^*, 22.51% ± 4.35%, *P* < 0.05, *n* = 3, mean ± SEM, 2-tailed Student’s *t* test). These data suggest that the progeroid mutation in *Lmna* causes defects of SuSCs, such that their number and osteogenic potential are compromised, leading to stem cell exhaustion and craniosynostosis.

### Association of the cytoskeleton with aging and suture patency in craniosynostosis.

**Figure 5:**
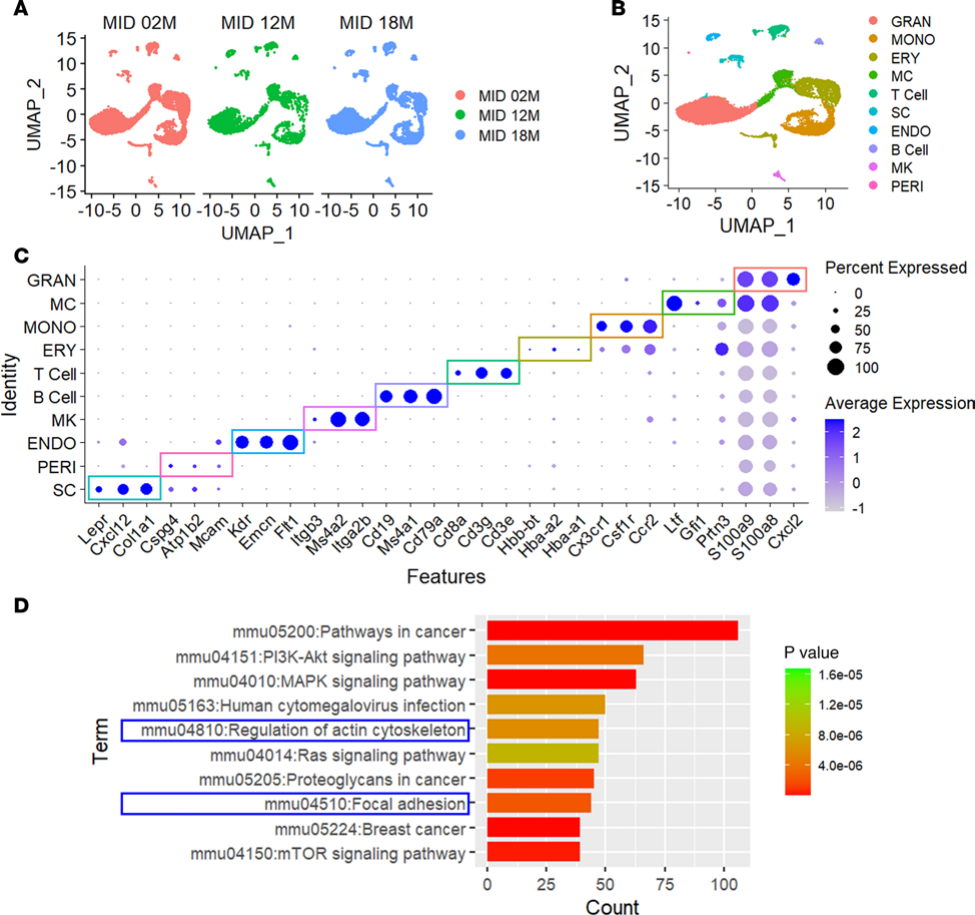
Transcriptomic profiling linking cytoskeletal regulation to skeletal cell aging. (**A**) UMAP plots visualize 3 batches of single-cell sequencing datasets. (**B**) Clustering a total of 20,811 cells into 10 subsets: granulocyte (GRAN), monocyte (MONO), erythrocyte (ERY), myeloid cell (MC), T lymphocyte (T Cell), skeletogenic cell (SC), endothelial cell (ENDO), B lymphocyte (B Cell), megakaryocyte (MK), and pericyte (PERI). (**C**) Dot plot showing marker gene expression for the classification of 10 clusters. (**D**) Pathway enrichment analysis of 2135 DEGs in SC cluster exhibited with a bar graph showing selected pathways, including cytoskeleton and cell adhesion, related to stem cell regulation from the top 10 enriched KEGG pathways (*P* < 0.05, log_FC_ < –0.1, 2M versus 18M).

To further decipher the regulatory processes linked to premature aging-mediated calvarial development and progeroid disorders, we took an unbiased approach. We processed the single-cell RNA-seq (scRNA-seq) raw datasets (Gene Expression Omnibus, GSE235176) available on GEO and performed additional analyses on midline (AF, PF, and SAG) sutures of mice at 2 months (2M), 12M, and 18M (37). Unsupervised clustering of transcriptomic profiles classified a total of 9,548 (2M), 3,280 (12M), and 8,053 (18M) cells by the Seurat R-package and subject to graph-based clustering using Uniform Manifold Approximation and Projection (UMAP) to visualize 10 distinct groups of cells (Figure 5A). They could be classified into 10 clusters, including the skeletogenic cell (SC) cluster for osteogenesis (Figure 5, B and C). In the SC cluster of 2,135 cells, the comparison of 2M and 18M differentially expressed genes (DEGs) identified selected pathways associated with aging using Kyoto Encyclopedia of Genes and Genomes (KEGG) as a reference database (*P* < 0.05, log_FC_ < –0.1). Two of the top 10 pathways were related to cell structure and organization: actin cytoskeleton and focal adhesion (Figure 5D). These suggest the likely importance of cytoskeleton structure, organization, and mechanosensation in skeletal cell aging and craniosynostosis.

### Effects of prelamin A accumulation on the nucleus and cytoskeleton.

**Figure 6:**
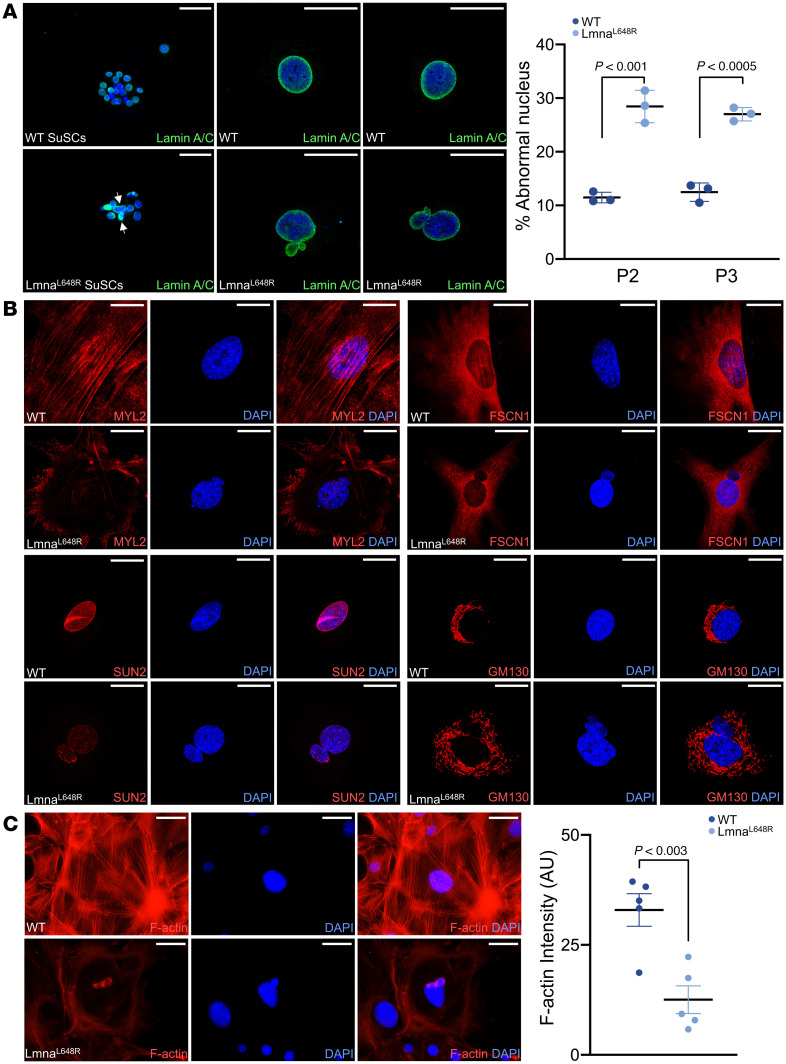
Cytoskeletal disruption by the progeroid accumulation of prelamin A. (**A**) Representative images of Lamin A/C staining examine nuclear morphology in the cultured SuSCs (left panels) and calvarial cells (2 right panels). Graphs show quantitation of the average percentage of calvarial cells containing abnormal nuclear shape in 3 independent experiments (*P* < 0.001 or 0.0005, *n* = 3, mean ± SD, 2-tailed Student’s *t* test). (**B**) Representative images showing immunostaining of phosphorylated myosin light chain 2 (MYL2), fascin1 (FSCN1), SUN2, and GM130 in the indicated cells. DAPI counterstaining in all images. (**C**) Representative images of filamentous actin (F-actin) staining examine the cytoskeleton of calvarial cells isolated from *Lmna^+/+^* (WT) and *Lmna^L648R/L648R^* (*Lmna^L648R^*) mice. Graphs indicate quantitation of the average intensity of F-actin in 5 independent experiments (*P* < 0.003, *n* = 3, mean ± SEM, 2-tailed Student’s *t* test). Scale bars: 50 μm (**A** and **C**), 20 μm (**B**).

The structural support of the cell nucleus requires the nuclear lamina, disruption of which by progeroid mutants, including *Lmna^L648R^*, is known to cause nuclear morphological abnormalities in fibroblast cells (2, 20). To examine if accumulations of farnesylated prelamin A, caused by the *Lmna^L648R^* mutation, also affect SCs, we isolated SuSCs from WT and mutant mice and established an ex vivo 3D culture capable of maintaining stem cell stemness (31, 38, 39). Consistent with prior observation in the fibroblast cells (20), immunostaining reveals misshapen nuclei in *Lmna^L648R^* but not WT SuSCs (Figure 6A). Abnormal nuclear morphologies were also found in mutant calvarial osteogenic cells with a significantly increased percentage of incidence (Figure 6A and Supplemental Figure 9; *P* < 0.001 or 0.0005, *n* = 3, mean ± SD, 2-tailed Student’s *t* test). Next, we analyzed perinuclear actin caps, which are dependent on lamin A/C and known to regulate the nuclear shape (40). The mutation disrupted actin cap lines as indicated by immunostaining of the actin interactor, phosphorylated myosin light chain 2 at Ser19 (Figure 6B and Supplemental Figure 10). Analysis of the actin-bundling protein fascin1 further revealed disruption of transmembrane actin-associated nuclear (TAN) lines, which formed predominantly in migrating cells (Figure 6B and Supplemental Figure 11). Calvarial osteoprogenitors tend to migrate after seeding, thus inducing polarization and formation of actin tubules to recruit fascin1. Since fascin1 also acts as a mechanotransducer of cytoplasmic forces, the results suggest impairment of nuclear deformation and movement during cell migration in *Lmna^L648R^* cells. The Linker of Nucleoskeleton and Cytoskeleton (LINC) complex, which links the lamina to cytosolic actin and microtubule filaments, was also disturbed in *Lmna^L648R^* calvarial osteogenic cells, as seen by disruption of SUN2 perinuclear staining (Figure 6B and Supplemental Figure 12). Moreover, direct examination of F-actin staining showed significant depolymerization in the mutant calvarial osteogenic cells (Figure 6C; *P* < 0.003, *n* = 5, mean ± SEM, 2-tailed Student’s *t* test). In addition, immunostaining of the Golgi marker GM130 revealed Golgi apparatus dispersal from the perinuclear into the cytoplasmic region (Figure 6B and Supplemental Figure 13), a process dependent on the actin cytoskeleton (41). Therefore, the results indicate that the LINC complex, as well as the cytoskeleton and nucleoskeleton organization, are severely interrupted by prelamin A with the L648R amino acid substitution. Together with bioinformatics studies in animals, our findings suggest an important role of nuclear-cytoskeletal dynamics in craniosynostosis of progeroid disorders.

### Actin polymerization in osteoblastogenesis of progeroid disorders.

**Figure 7:**
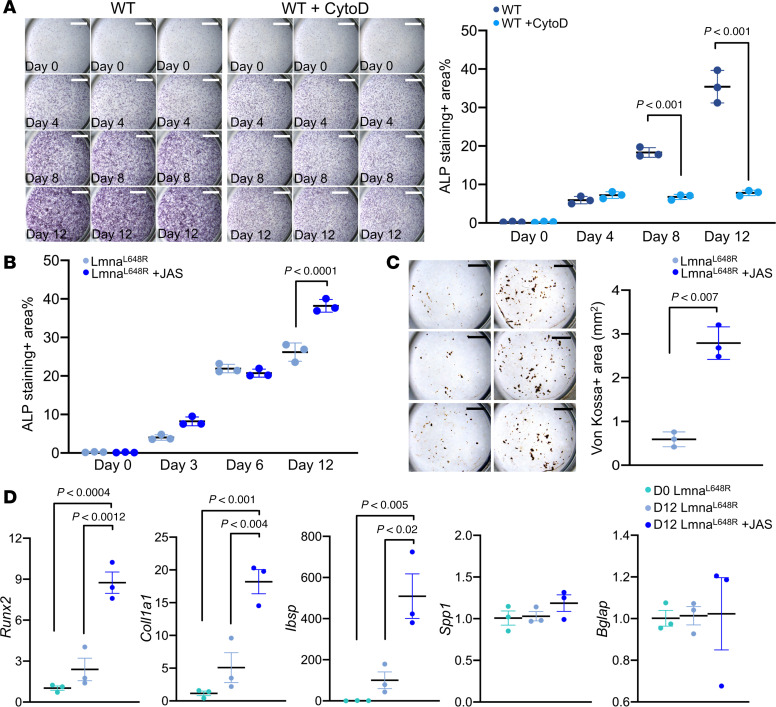
Effects of actin polymerization on osteogenic defects associated with the *Lmna^L648R^* mutation. (**A**) Alkaline phosphatase staining examining osteoblast differentiation of calvarial cells in ex vivo culture without or with CytoD at day 0, 4, 8, and 12. The graph shows the inhibitory effects of CytoD on osteoblast differentiation (*P* < 0.001, *n* = 3, mean ± SD, 1-way ANOVA test with Tukey’s multiple comparisons). (**B**) The graph is representative of 3 independent experiments analyzing the alkaline phosphatase (ALP) activity of *Lmna^L648R^* calvarial cells cultured in differentiation media without or with JAS for days as indicated (*P* < 0.0001, *n* = 3, mean ± SD, 1-way ANOVA test with Tukey’s multiple comparisons). (**C**) Representative images of von Kossa staining examine mineralized nodule formation in the culture of *Lmna^L648R^* calvarial cells without or with JAS for 21 days. Graphs show the quantitation of the average stained area in 6 independent experiments (*P* < 0.007, *n* = 3, mean ± SEM, 2-tailed Student’s *t* test). (**D**) Plots show qPCR results examining the expression of osteoblast markers after culture of mutant cells in differentiation media without or with JAS for 12 days (*P* < 0.0012, < 0.004, < 0.02, *n* = 3, mean ± SEM, 1-way ANOVA test with Tukey’s multiple comparisons). Scale bars: 3 mm (**A** and **C**).

**Figure 8:**
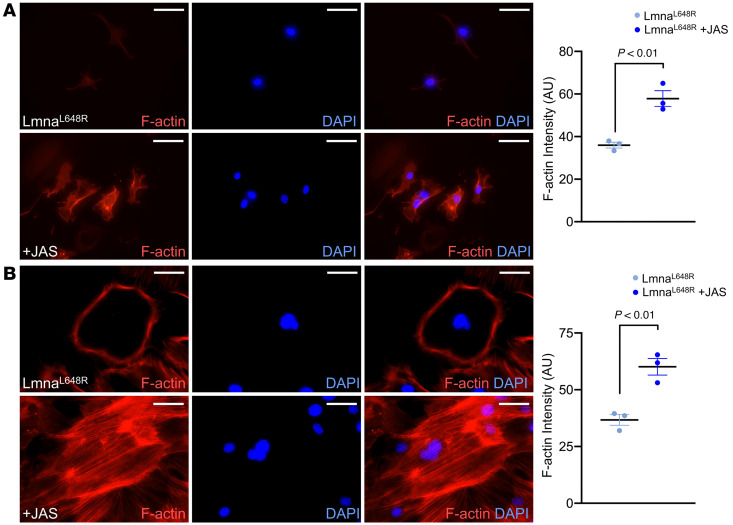
JAS promotes actin polymerization in osteogenic cells. (**A**) Representative images of F-actin staining, analyzing the cytoskeleton of *Lmna^L648R^* suture stem cells without or with JAS treatment. Graphs show the quantification of the average intensity of F-actin in five independent experiments (*P* < 0.01, *n* = 3, mean ± SEM, 2-tailed Student’s *t* test). (**B**) Representative images of F-actin staining examine the cytoskeleton of *Lmna^L648R^* calvarial cells without or with JAS treatment. Graphs show the quantification of the average intensity of F-actin in 5 independent experiments (*P* < 0.01, *n* = 3, mean ± SEM, 2-tailed Student’s *t* test). Scale bars: 50 μm (**A** and **B**).

To determine the role of actin dynamics in calvarial morphogenesis and craniosynostosis, we examined osteoblastogenesis regulation by the cytoskeleton in calvarial osteogenic cells (42). Inhibition of actin polymerization by cytochalasin D (CytoD) significantly reduced their differentiation into calvarial osteogenic cells (Figure 7A; *P* < 0.001, *n* = 3, mean ± SD, 1-way ANOVA with Tukey’s multiple comparisons). Jasplakinolide (JAS) is an agonist that stabilizes F-actin, thus significantly enhancing actin polymerization in both *Lmna^L648R^* suture stem (Figure 8A and Supplemental Figure 14A) and calvarial osteoprogenitor (Figure 8B and Supplemental Figure 14B) cells (Figure 8; *P* < 0.01, *n* = 3 per group, mean ± SEM, 2-tailed Student’s *t* test). Although expression of *Actb*, which encodes β-actin, was reduced in the differentiated mutant osteoblasts (Supplemental Figure 15), expression of *Actg1*, encoding γ-actin, remained unchanged in the mutant cells (Supplemental Figure 15). Thus, reduced F-actin intensity in the mutant cells may be partly attributed to transcriptional alteration. However, JAS treatment markedly increased F-actin intensity (Figure 8) and restored impaired osteoblast differentiation and mineralization (Figure 7, B–D). Therefore, these findings indicate that defects in actin polymerization, rather than changes in actin gene expression, underlie the reduced F-actin intensity and impaired osteoblast differentiation in Lmna^L648R^ mutant cells. Next, we tested whether actin polymerization can alleviate the osteogenic defects caused by the progeroid mutation. The addition of JAS to the ex vivo culture of mutant calvarial cells significantly increased their differentiation into osteoblast cells (Figure 7B; *P* < 0.0001, *n* = 3, mean ± SD, 1-way ANOVA test with Tukey’s multiple comparisons). In cultured cells from *Lmna^L648R^* mice, JAS also significantly elevated mineralized nodule formation (Figure 7C; *P* < 0.007, *n* = 3, mean ± SEM, 2-tailed Student’s *t* test). We then examined the expression of osteogenic genes critical for osteoblast differentiation. Total RNA isolated from the cells of *Lmna^L648R^* mice before (day 0, D0) and after 12 days (D12) culture in differentiation media, without or with the presence of JAS, was analyzed by qPCR (Figure 7D). The results indicate significant enhancements of early-mid differentiation markers *Runx2*, *Col1a1*, and Integrin Binding Sialoprotein (*Ibsp*), but not mature osteoblast markers *Spp1*/OPN and *Bglap*/OCN, in the mutant cells by JAS (Figure 7D; *P* < 0.0012, < 0.004, < 0.02 as indicated, *n* = 3 per group, mean ± SEM, 1-way ANOVA test with Tukey’s multiple comparisons). Our data suggest that defects in osteoblast cells occurring in a progeroid disorder can be alleviated through the modulation of cytoskeletal dynamics. The findings provide a functional connection between the nuclear-cytoskeletal organization and craniofacial deformities by the accumulation of permanently farnesylated prelamin A, resulting from a progeroid disease.

## Discussion

This study presents compelling evidence for craniosynostosis caused by the mutation of *Lmna*, which disrupts prelamin A processing and causes progeroid phenotypes. The accumulation of permanently farnesylated prelamin A leads to reduced cell proliferation and differentiation in osteogenic cells, leading to decreases in mineralization. As a result, aberrant suture fusion occurs under low bone density, in contrast to excessive ossification in conventional synostosis (28, 43). These features, resembling SuSC-specific disruption of BMPR1A (31), are causatively linked to stem cell deficiency. Intrasutural bones detected in *Bmpr1a* mutant mice are reminiscent of Womian bones prominently present in patients with progeroid syndrome — e.g., HGPS, and MAD-B (33–35). These are caused by mutations in *LMNA* and *ZMPSTEM24*, respectively, that — like the *Lmna^L648R^* mutation investigated here — also impair prelamin A processing. Our findings indicate that stem cell alterations result from the accumulation of farnesylated prelamin A, supporting SuSC exhaustion as an underlying mechanism for suture synostosis. The loss of stem cells in the mutants is a pathogenic process that progresses over time, and suture fusion follows a similarly temporal pattern. To accurately assess stem cell frequency, it is essential to perform the analysis before SuSCs are completely depleted following synostosis. Therefore, it is important to capture the dynamic changes in stem cell populations between WT and mutant sutures at stages before the disappearance of SuSCs in synostosed sutures (31).

Progeroid *Lmna^L648R^* mutation in osteogenic cells causes defects in nuclear morphology, cytoskeleton and nucleoskeleton organization, and the LINC complex. Thus, our findings indicate an essential role for the connections between nuclear lamina and cytoskeletal filaments in osteoblast differentiation, possibly due to altered mechanosensation. Furthermore, inhibition of actin polymerization represses osteoblast differentiation. Stabilization of actin polymers with an agonist successfully alleviates deficiencies in osteogenesis and mineralization caused by progeroid *Lmna^L648R^* mutation. It would be of interest to determine whether SuSCs are similarly affected in other progeroid models, as actin dynamics also mediate osteogenic functions. Alterations in cytoskeleton organization and nucleoskeleton assembly are also associated with other skeletal diseases. Autosomal dominant mutations in *FLNM* encoding filamin B, an actin-binding cytoskeletal protein, cause Larsen syndrome with a distinct craniofacial appearance, and Piepkorn osteochondrodysplasia exhibiting craniosynostosis (44, 45). A recessive loss-of-function mutation of *FLNM* results in spondylocarpotarsal synostosis syndrome (46). Therefore, the dynamics of the nuclear-cytoskeletal organization and assembly may be generally important for osteoblastogenesis and healthy craniofacial development.

The progeroid *Lmna^L648R^* mutation disrupts the perinuclear actin cap and TAN lines, as well as the LINC complex. These are critical components associated with mechanotransduction in osteoblastogenesis (47). The LINC complex plays an important role in mechanosensing, transmitting forces from the extracellular matrix into the nucleus (48). The LINC complex and actin cap regulate the cellular cytoskeleton in osteogenesis through modulation of Hippo and Wnt pathways (49, 50). A variety of developmental processes require these pathways signaling via Yap-Taz and β-catenin, whose disruption results in skeletal diseases (51–53). Hippo signaling mediated by Yap-Taz is critical for mechanosensing and mechanotransduction in the cytoskeletal response to extracellular stimuli (54). Nucleus-cytoskeleton interactions play a crucial role in remodeling the extracellular matrix to regulate SSC fate determination mediated by Wnt/β-catenin signaling (50). Our findings also support previous notions on the loss of mechanotransduction caused by the disruption of nucleus-cytoskeleton interactions, leading to a round-flat cell shape in progeroid and physiological aging (55). Deciphering the downstream effects of the nucleus-cytoskeleton interplay on osteogenic cell types promises further advancements in our fundamental knowledge and potential therapeutics for accelerated and physiological skeletal aging.

## Methods

### Sex as a biological variable.

Both male and female mice were used in this study, and similar findings are reported for both sexes.

### Animals.

The *Lmna^L648R^* (Jackson Laboratory, strain no. 037310) and *Axin2^mGFP^* (Jackson Laboratory, strain no. 037313) mouse strains and genotyping methods were reported previously (20, 56). The *Lmna*^+/+^ (WT) and *Lmna^L648R/L648R^* homozygous mice used in the experiments are > 90% pure C57BL/6J (Jackson Laboratory, strain no. 000664) background. For μCT analysis, mouse skulls were scanned at 6 μm or 10 μm resolution by a high-resolution 3D x-ray microscope, Skyscan 1271 model (Bruker), followed by imaging analysis with AMIRA software (Thermo Fisher Scientific) as described previously (31). The renal capsule transplantation of freshly isolated cells from the suture mesenchyme, as well as the limiting dilution analysis for the determination of stem cell frequency, were performed based on previously published protocols (38, 39, 57).

### Cells.

Primary suture mesenchymal cells containing SuSCs were isolated from mouse calvaria as described (31, 38, 39). For cell exposure, an approximately 1.5 mm–wide calvarium containing the sagittal suture and its adjacent parietal bones was dissected, followed by the separation of the parietal bone segments. Next, the samples were incubated with 0.2% collagenase in PBS at 37°C for 1.5 hours. The dissociated cells were filtered through a 40 mm strainer, followed by resuspension in DMEM media for transplantation analysis, in DMEM containing 5% FBS for cell sorting or in supplemented media for ex vivo sphere culture (38, 39, 57). The isolation of primary calvarial cells was performed as described previously (29). The isolated cells were cultured in αMEM containing 10% FBS and 1% penicillin/streptomycin. For analysis of actin dynamics, cells were cultured without or with the presence of 0.5 μM CytoD (C8273, MilliporeSigma) and JAS (CAS 102396-24-7, Santa Cruz Biotechnology). To examine cell proliferation, BrdU was included in the culture media for 12 hours, followed by fixation with a 1:1 acetone/methanol solution for 2 minutes at room temperature (29). The detection of BrdU was performed according to the manufacturer’s protocol (Thermo Fisher Scientific). For osteoblast differentiation, cells were cultured in differentiation media containing 50 μg/mL ascorbic acid and 4 mM β-glycerophosphate (29). Alkaline phosphatase staining was conducted using 1-Step NBT/BCIP Substrate Solution according to the manufacturer’s protocol (Thermo Fisher Scientific). The positively stained areas were analyzed with ImageJ software (NIH) for quantification.

### Histology and staining analyses.

Sample preparation, fixation, and embedding in paraffin were performed as described (26, 32, 58, 59). Using a microtome (Epredia-HM 325, Thermo Fisher Scientific), paraffin sections were prepared and stained with H&E for histological evaluation as described previously (29, 60, 61). For immunostaining, antigen retrieval was carried out before incubation with primary and secondary antibodies (29, 32, 62), followed by an avidin/biotinylated complex–based detection method using a DAB Substrate Kit or biotin-streptavidin fluorescent conjugates (H3300, PK-6100, SK-4100, and SA-5001-1, Vector Laboratories) with DAPI counterstaining (H-1800, Vector Laboratories). For analysis of spheres, the cultures were collected onto the glass slides by cytospin (Cytospin 4, Thermo Fisher Scientific), followed by fixation with 4% paraformaldehyde in PBS for 10 minutes at room temperature (31). The cultured sphere cells were permeabilized with 0.5% Triton X-100 for 5 minutes, followed by blocking with 5% BSA for immunostaining (31). Mouse monoclonal antibodies against Bmpr1a (NBP2-37421, 1:75, Novus Biologicals), fascin1 (SC-21743, 1:100, Santa Cruz), and Alexa Fluor 568 phalloidin (A12380, 1:400, Invitrogen); rabbit polyclonal antibodies Osx (ab22552, 1:800, Abcam), OCN (23418-1-AP, 1:50, Proteintech), Gli1 (NBP1-78259, 1:100, Novus Biologicals), Myl2 (3671, 1:100, Cell Signaling Technology), and GM130 (2296, 1:200, Cell Signaling Technology); rabbit monoclonal antibodies lamin A/C (MA5-35284, 1:200, Invitrogen) and SUN2(EPR6557, 1:100, Abcam); anti-rabbit secondary antibody (BA-1000, 1:200, Vector laboratories); and anti-mouse IgG secondary antibodies (PK-2200, 1:250, Vector laboratories), were used in the immunostaining studies. Images were captured with a Nikon Eclipse TS2 microscope (Nikon), a Zeiss Axio Observer microscope (Carl Zeiss), or a Zeiss LSM880 confocal microscope (Carl Zeiss) imaging system. For apoptosis, paraffin sections were analyzed by TUNEL staining using ApopTag Plus In Situ Apoptosis Fluorescein Detection Kit (S7111, MilliporeSigma) according to the manufacturer’s protocol. The TUNEL^+^ cells were quantified using ImageJ as described (26, 32, 61). To determine the bone formation rate, alizarin red and calcein double labeling analysis was performed as previously described (63). Briefly, 2-month-old mice received i.p. injections of calcein (10 mg/kg; C0875, MilliporeSigma), followed 14 days later by alizarin red (30 mg/kg; A5533, MilliporeSigma). Calvariae were then processed for cryosectioning (CM1950, Leica Biosystems) and fluorescence imaging using a Zeiss Axio Observer microscope. The distance between the 2 fluorescent labels was measured at 6 evenly spaced sites per sample using ImageJ software. The bone formation rate per bone surface (BFR/BS, μm^3^/μm^2^/day) was calculated as BFR/BS = MAR × (MS/BS/100), where MS/BS represents the mineralizing surface per bone surface.

### Quantitative gene expression analysis.

The isolation of total RNA was performed using the mirVana kit (Thermo Fisher), followed by the generation of cDNA using iScript Reverse Transcription Supermix (Bio-Rad). The cDNA products were subjected to real-time PCR analysis using specific forward and reverse primer sets listed below. The PCR reactions were carried out with an initial denaturation at 95°C for 5 minutes, followed by 39 amplification cycles of 95°C for 15 seconds, 61°C for 30 seconds, and 72°C for 30 seconds. GAPDH was used as an internal control for data normalization, followed by quantification using the 2^–ΔΔCT^ method. Primer sequences are provided in Table 2.

### scRNA-seq and bioinformatics.

Raw datasets for scRNA-seq (Gene Expression Omnibus, GSE235176) of mouse midline (AF, PF, and SAG) sutures at 2, 12, and 18 months were processed and analyzed using Seurat version 5.0.2 on R software version 4.3.1. The datasets were first filtered by the parameter of cells with more than 200 genes detected and genes detected in more than 3 cells for subsequent analyses. Three Seurat objects were then created and normalized, followed by integration for 2-month-old, 12-month-old, and 18-month-old samples. Dimension reduction was performed using the first 20 principal components with UMAP, and cell clustering was set at “resolution = 0.1.” A graph-based clustering then classified cells into clusters, which relied on an algorithm based on weighted shared nearest neighbor modularity optimization. Marker genes for each cluster were identified with the Wilcoxon rank-sum test with default parameters via the “FindAllMarkers” function of Seurat software. Next, DEGs from the 2-month and 18-month datasets were filtered by log_2_ fold change of less than 0.1. The DEGs with a *P* < 0.05 adjusted by Benjamini-Hochberg’s method were considered to be significant. The processed dataset was integrated using the IntegrateData function in the R package Seurat. The enriched DEGs for the KEGG function in the SC cluster were then analyzed using the clusterProfiler in R software, or input into online platforms of DAVID (v2023q1, https://davidbioinformatics.nih.gov/).

### Statistics.

Statistical analyses were performed using Microsoft Excel 2021 and GraphPad Prism 8. The normality of data distribution was validated using the Shapiro-Wilk normality test. Significance was assessed by a 2-tailed Student’s *t* test for comparisons between 2 groups or a 1-way ANOVA for comparisons among more than 2 groups. When ANOVA indicated significant differences, Tukey’s post hoc test was used for multiple comparisons. All data were presented as mean ± SD or mean ± SEM, with a *P* value less than 0.05 considered statistically significant.

### Study approval.

IACUC at the ADA Forsyth Institute approved the care and use of experimental animals described in this work.

### Data availability.

All data needed to evaluate the conclusions in the paper are present in the paper and the Suporting Data Values file. Data for single-cell transcriptomics of age-related changes in murine cranial sutures (GSE235176) are available for interactive analysis on Gene Expression Omnibus. The *Lmna^L648R^* mouse strain is available at Jackson Laboratory (strain no. 037310). The code used for sequencing data analysis and visualization is available on GitHub (https://github.com/whsulab/Lmna; commit ID 7172059fda8c73b7eba1a92016acde8312512e32).

## Author contributions

KL and TH are co–first authors who contributed equally to this work. The amount and significance of the work determine authorship order. KL, TH, HU, and WH conceived and designed the experiments and analyzed the data. KL, TH, and WH wrote the paper. TW, SM, and HW contributed to data interpretation, providing the *Lmna* mutant mouse strain and suggestions on manuscript preparation. WH, SM, and HW secured funding support.

## Funding support

This work is the result of NIH funding and is subject to the NIH Public Access Policy. Through acceptance of this federal funding, the NIH has been given the right to make the work publicly available in PubMed Central.

- National Institute of Dental and Craniofacial Research and National Institute on Aging of the NIH.
- R01DE015654 and R01DE026936 to WH.
- R01AG075047 to SM, HW, and WH.
- NIH S10OD034405.

## Supplementary Material

Supplemental data

Supporting data values

## Figures and Tables

**Table 1:**
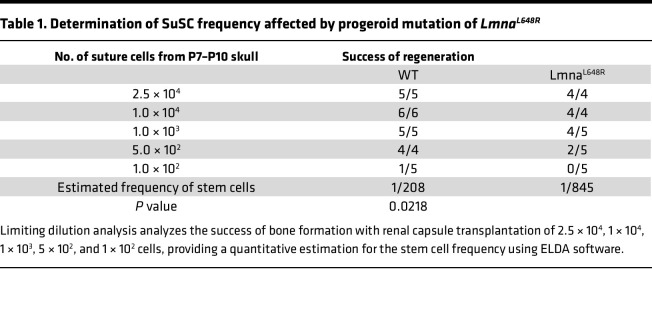
Determination of SuSC frequency affected by progeroid mutation of *Lmna^L648R^*

**Table 2:**
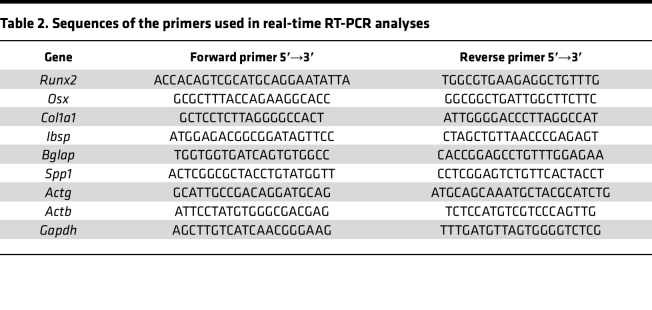
Sequences of the primers used in real-time RT-PCR analyses

## Version 1 02/03/2026

In-Press Preview

## Version 2 03/09/2026

Electronic publication
