# Supplementary material for: Stem cell–associated osteogenic deficiency causes craniofacial deformities with progeroid accumulation of prelamin A
Source: JCI Insight. 2026 Feb 3;11(5):e196932. doi: 10.1172/jci.insight.196932 (PMC13041684; doi:10.1172/jci.insight.196932)
Supplement: Supplemental data [file jciinsight-11-196932-s171.pdf]

Supplementary Materials for

Stem cell-associated osteogenic deficiency causes craniofacial deformities with progeroid accumulation of prelamin A

Kai Li[1†], Trunee Hsu[1,5†], Hitoshi Uchida[1‡], Tingxi Wu[1], Susan Michaelis[6], Howard Worman[7], and Wei Hsu[1, 2, 3, 4]*

*Corresponding author. Email: whsu@forsyth.org; weh437@med.harvard.edu

This PDF file includes:

Supplemental Figure 1. Multiple suture synostosis in *Lmna*^{L648R} mice at 7 months of age.

Supplemental Figure 2. Craniosynostosis in the 3-month-old *Lmna*^{L648R} mutant.

Supplemental Figure 3. Histological evaluation of suture fusion in the *Lmna*^{L648R} mice.

Supplemental Figure 4. Proliferation of calvarial cells decreased by the *Lmna*^{L648R} mutation.

Supplemental Figure 5. Reduction of osteoprogenitor cells in the WT and *Lmna*^{L648R} cranial sutures.

Supplemental Figure 6. Defective osteoblast differentiation and mineralization in premature aging.

Supplemental Figure 7. No effect of *Lmna*^{L648R} mutation on apoptosis.

Supplemental Figure 8. Effects of the *Lmna*^{L648R} mutation on stem cells in cranial sutures.

Supplemental Figure 9. Nuclear morphologies of WT and *Lmna*^{L648R} calvarial cells.

Supplemental Figure 10. Perinuclear actin cap affected by the *Lmna*^{L648R} mutation.

Supplemental Figure 11. Defective assembly of TAN lines in *Lmna*^{L648R} mutant cells.

Supplemental Figure 12. Disruption of the LINC complex in *Lmna*^{L648R} mutant cells.

Supplemental Figure 13. *Lmna*^{L648R} mutation causes Golgi dispersal.

Supplemental Figure 14. JAS promotes actin polymerization in *Lmna*^{L648R} osteogenic cells.

Supplemental Figure 15. The effect of JAS on actin gene expression in *Lmna*^{L648R} osteogenic cells.

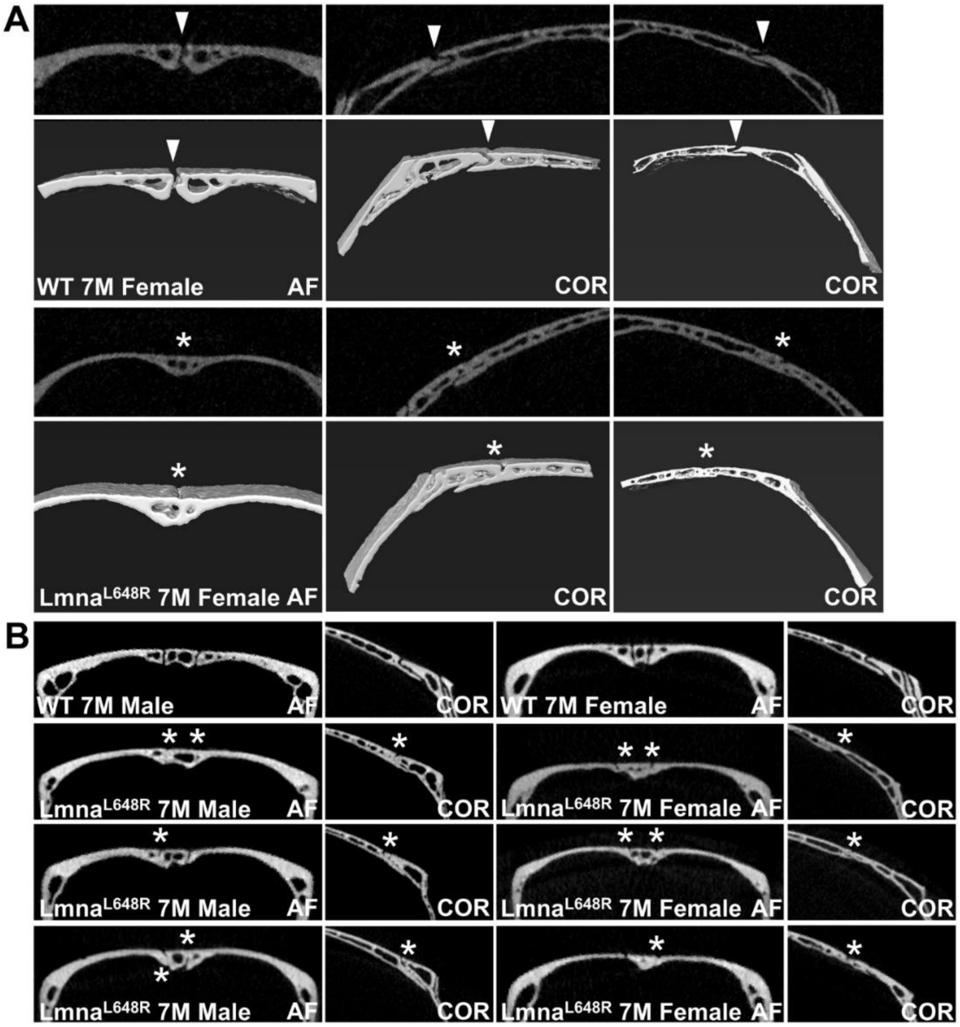

Supplemental Figure 1. Multiple suture synostosis in *Lmna*^{L648R} mice at 7 months of age. **(A)** Representative µCT-scanned coronal sections and 3D renderings of *Lmna+/+* (WT) and *Lmna*$^{L648R/L648R}$ (*Lmna*^{L648R}) female skulls at 7 months. **(B)** Images of µCT scanned three different 7-month-old *Lmna*^{L648R} males and females showing synostosed sutures as indicated by asterisks. AF, anterior frontal; COR, coronal.

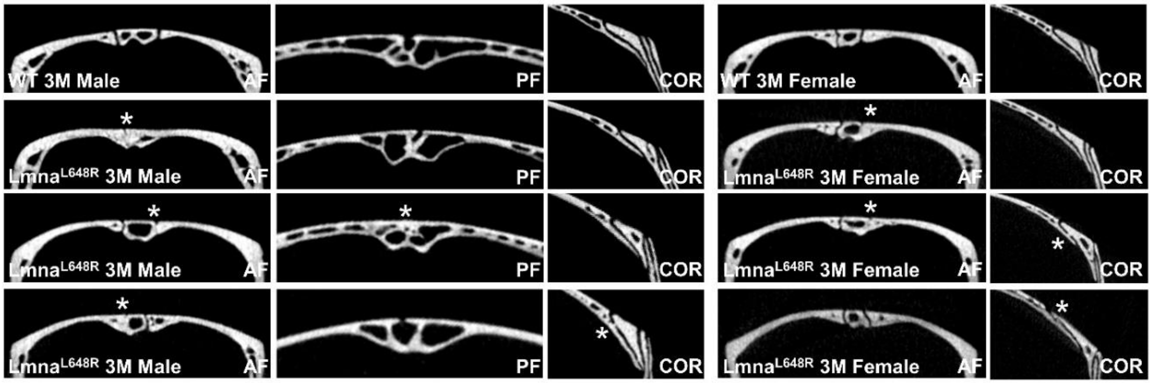

Supplemental Figure 2. Craniosynostosis in the 3-month-old *Lmna*^{L648R} mutant. Images of µCT-scanned 3-month-old WT and three different *Lmna*^{L648R} males and females, analyzing aberrant suture fusion indicated by the asterisk. AF, anterior frontal; COR, coronal; PF, posterior frontal.

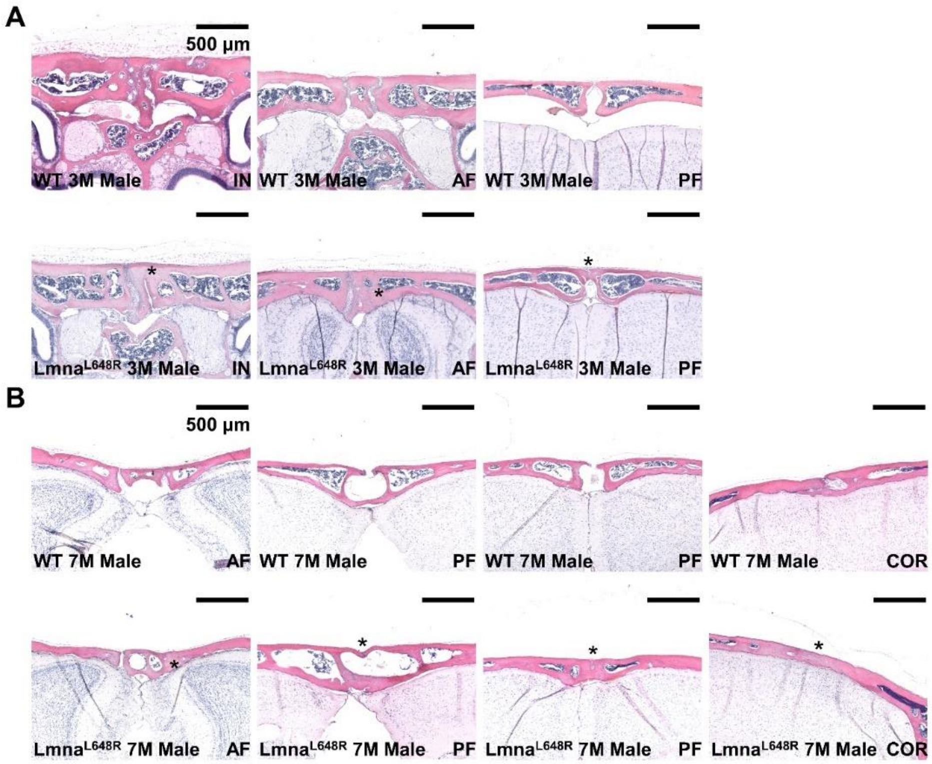

Supplemental Figure 3. Histological evaluation of suture fusion in the *Lmna^{L648R}* mice. Hematoxylin and eosin staining of 3-month-old **(A)** and 7-month-old **(B)** WT and *Lmna^{L648R}* calvarial sections. Asterisks indicate synostosed sutures. AF, anterior frontal; COR, coronal; IN, internasal; PF, posterior frontal. Scale bars, 500 μm.

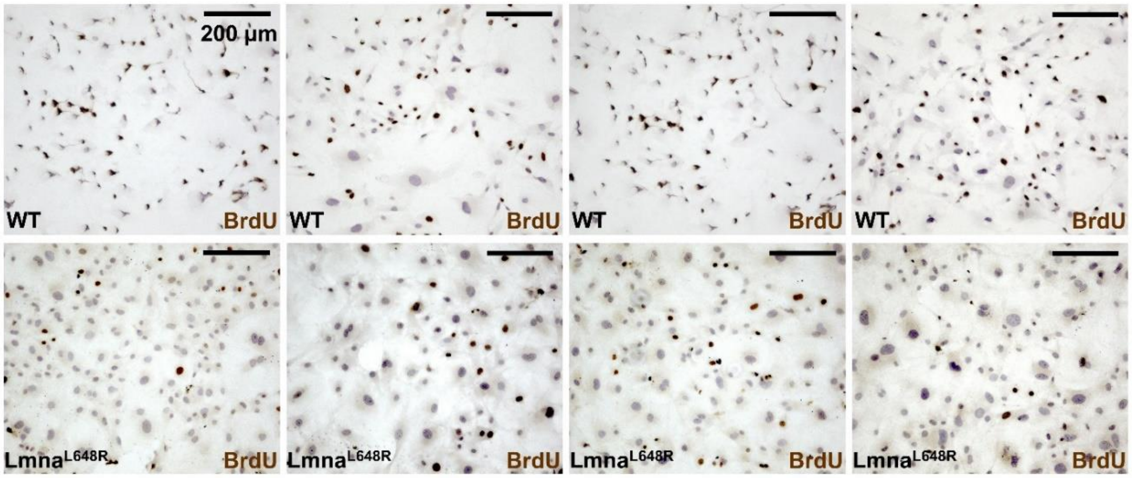

Supplemental Figure 4. Proliferation of calvarial cells decreased by the *Lmna*^{L648R} mutation. Images showing the representative field of views for calvarial cells positive for transient BrdU labeling analysis in the cultured WT and *Lmna*^{L648R} calvarial cells. The average percentage of BrdU-positive cells is then obtained for the quantification study shown in Fig. 2A. Scale bars, 200 μm.

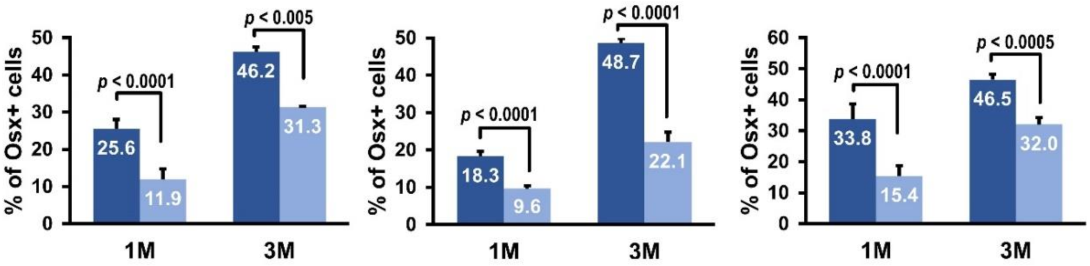

Supplemental Figure 5. Reduction of osteoprogenitor cells in the WT and *Lmna*^{L648R} cranial sutures. Graphs indicate the quantitation of osteoprogenitor cells present in the 1-month and 3-month WT and *Lmna*^{L648R} cranial sutures (1M, 3M). The average percentage of Osx-positive cells was analyzed by the immunostaining of the WT and *Lmna*^{L648R} cranial sutures in three independent experiments (two-sided Student's *t*-test, mean ± SD).

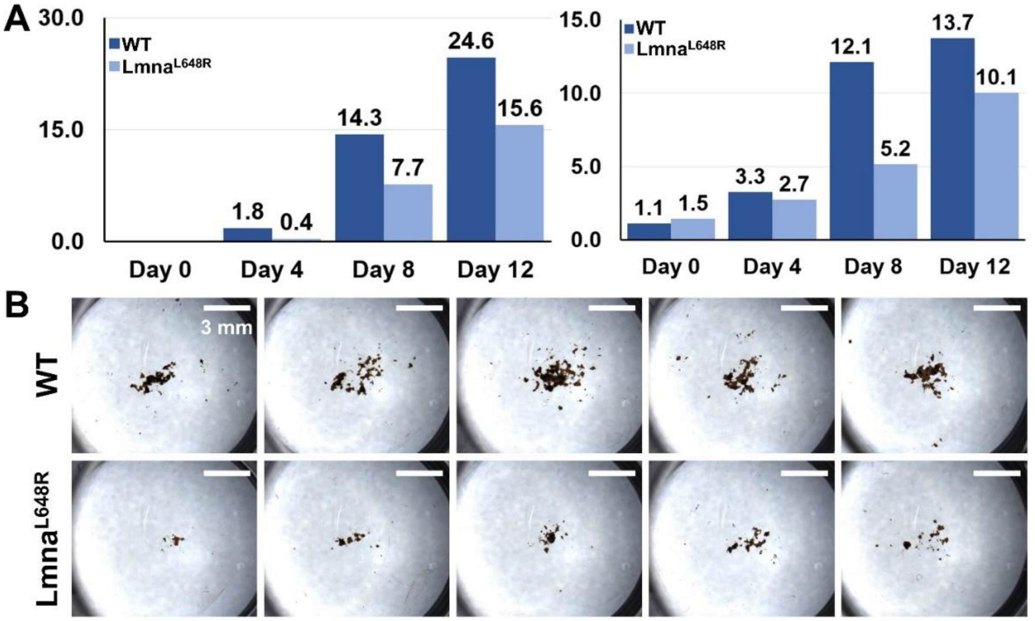

Supplemental Figure 6. Defective osteoblast differentiation and mineralization in premature aging. (A) Graphs show two of three independent experiments analyzing the alkaline phosphatase (ALP) activity of WT and *Lmna*^{L648R} calvarial cells cultured in differentiation media for days as indicated. **(B)** Images of von Kossa staining show five independent experiments examining mineralized nodule formation in the culture of WT and *Lmna*^{L648R} calvarial cells for 21 days. Scale bars, 3 mm.

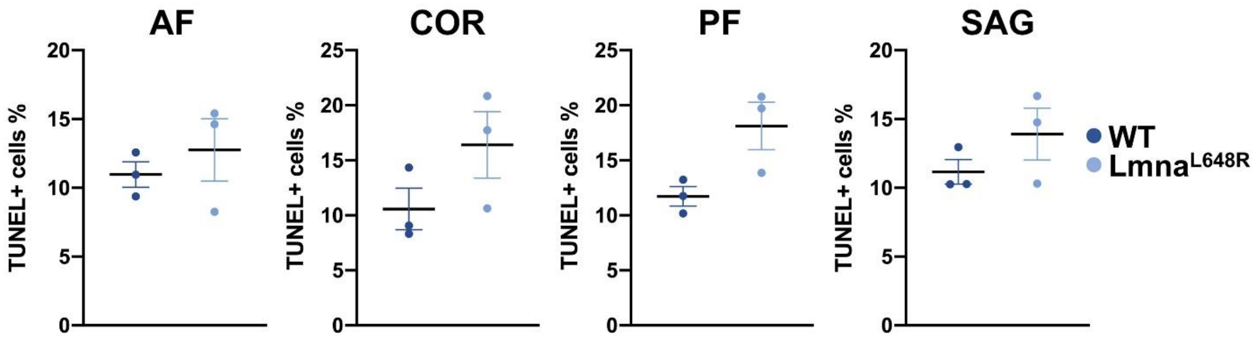

Supplemental Figure 7. No effect of *Lmna^{L648R}* mutation on apoptosis. Graphs indicate the percentage of TUNEL+ cells in the 3-month-old WT and *Lmna^{L648R}* sutures. AF, anterior frontal; COR, coronal; PF, posterior frontal; SAG, sagittal.

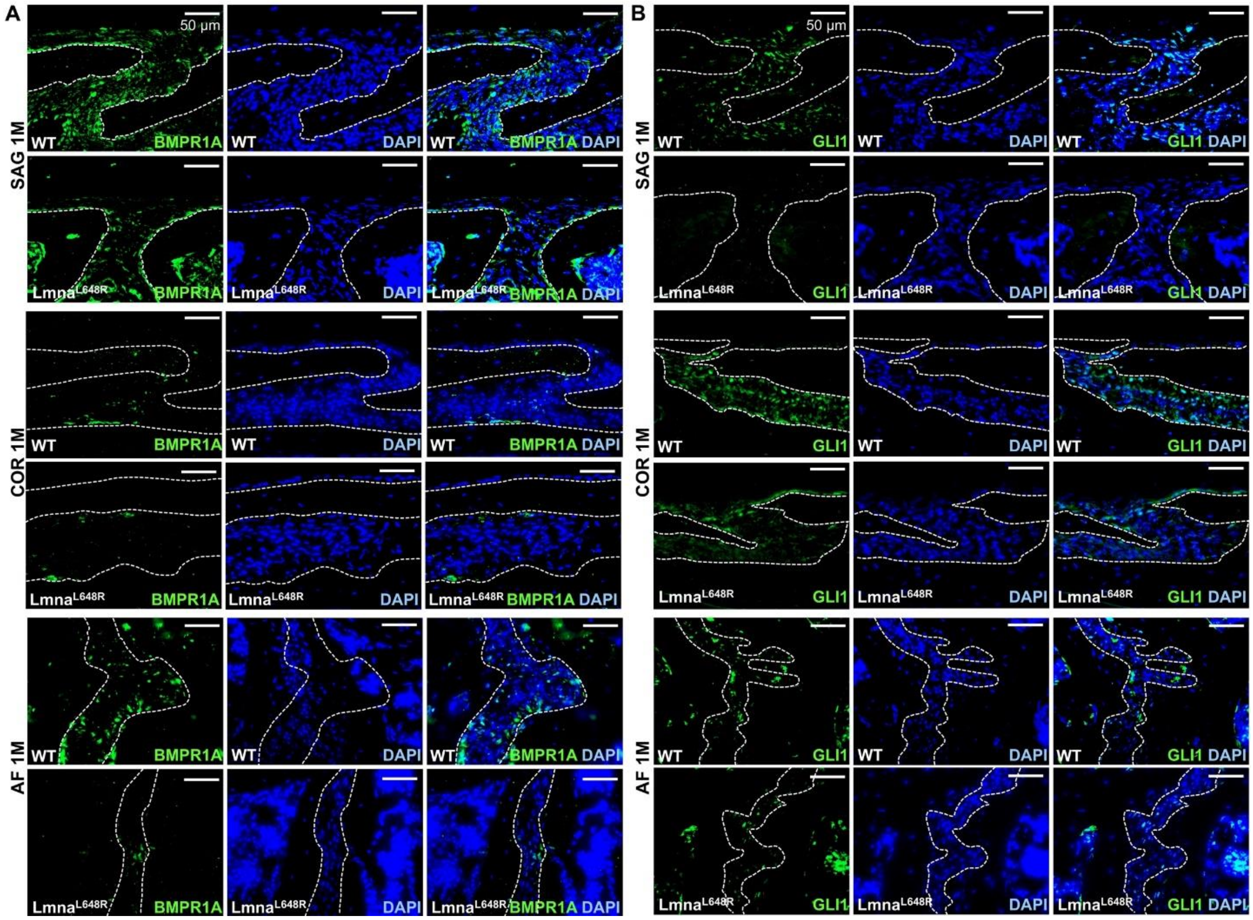

Supplemental Figure 8. Effects of the *Lmna^{L648R}* mutation on stem cells in cranial sutures. Immunostaining images showing cells expressing suture stem cell markers, BMPR1A+ **(A)** and GLI1+ **(B)** cells, within the indicated 1-month-old (1M) sutures. AF, anterior frontal; COR, coronal; SAG, sagittal. Scale bars, 50 µm.

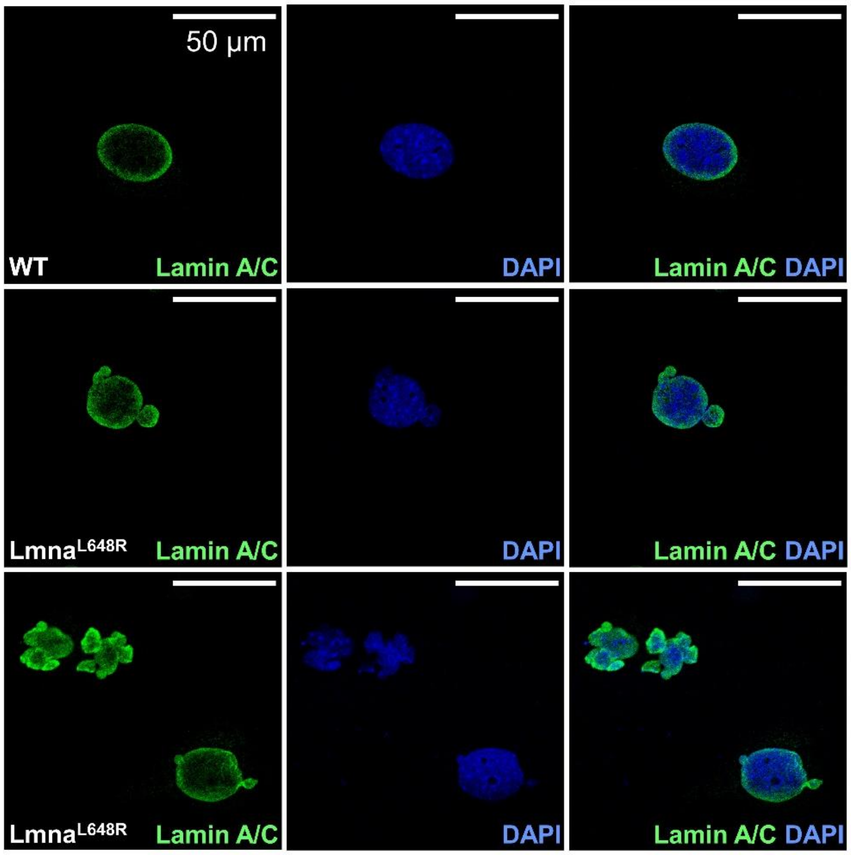

Supplemental Figure 9. Nuclear morphologies of WT and *Lmna^{L648R}* calvarial cells. Images of Lamin A/C staining show representative normal and abnormal nuclear morphologies in cultured calvarial cells. Scale bars, 50 µm.

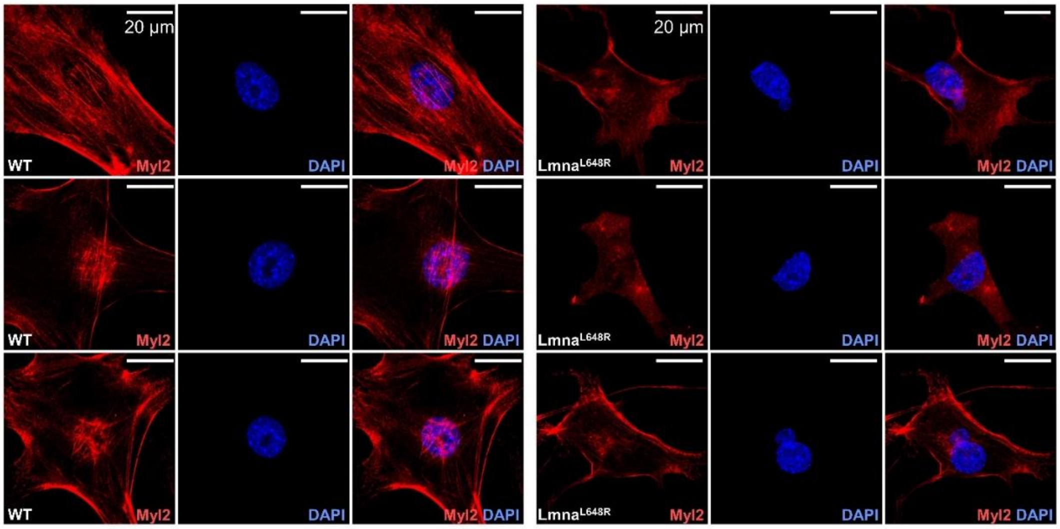

Supplemental Figure 10. Perinuclear actin cap affected by the *Lmna*^{L648R} mutation.
Immunostaining of phosphorylated myosin light chain 2 at Ser19 (Myl2), examining perinuclear actin cap fibers in WT and *Lmna*^{L648R} calvarial cells. Scale bars, 20 µm.

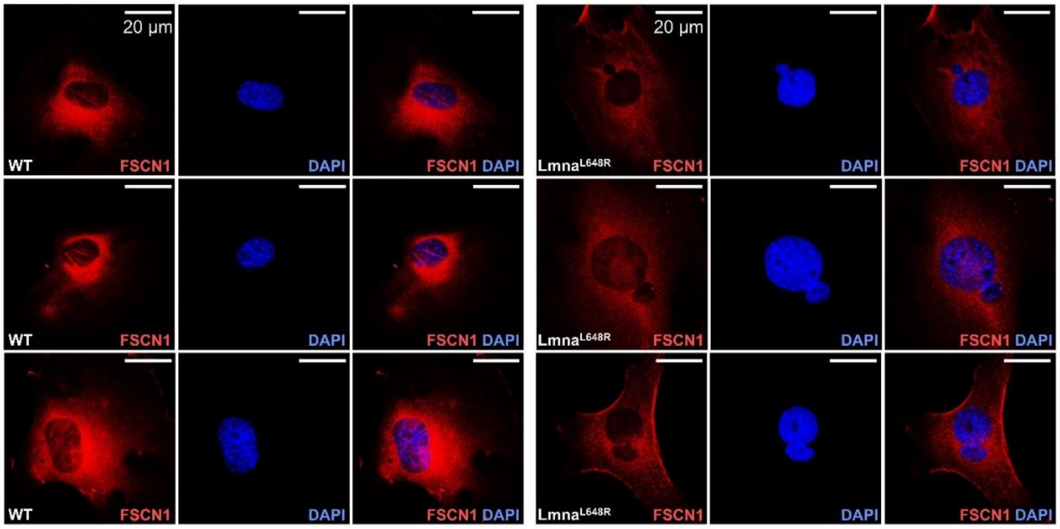

Supplemental Figure 11. Defective assembly of TAN lines in *Lmna*^{L648R} mutant cells. Immunostaining of FSCN1 examining Transmembrane Actin-Associated Nuclear (TAN) lines in WT and *Lmna*^{L648R} calvarial cells. Scale bars, 20 μm.

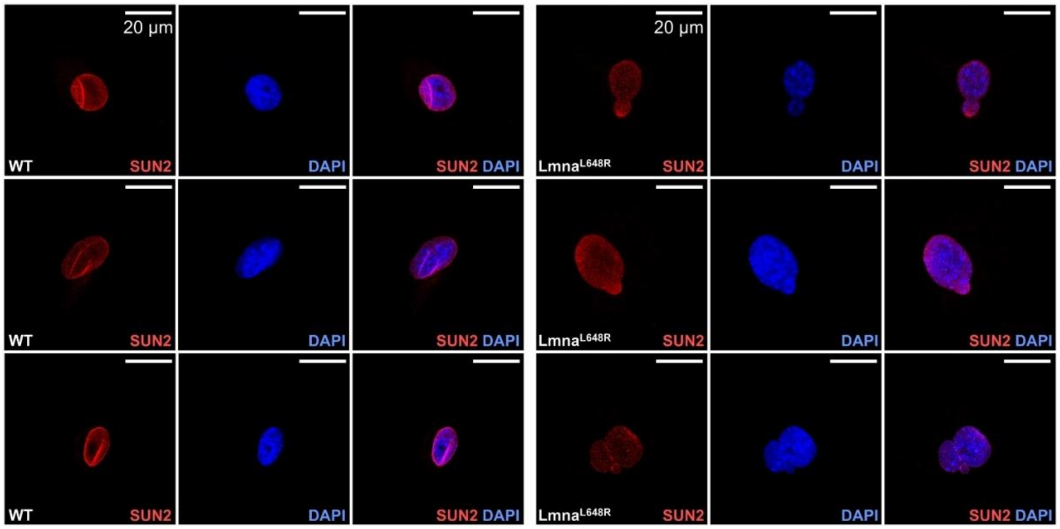

Supplemental Figure 12. Disruption of the LINC complex in *Lmna^{L648R}* mutant cells. Examination of SUN2 residing in the inner nuclear membrane of WT and *Lmna^{L648R}* calvarial cells by immunostaining. Scale bars, 20 μm.

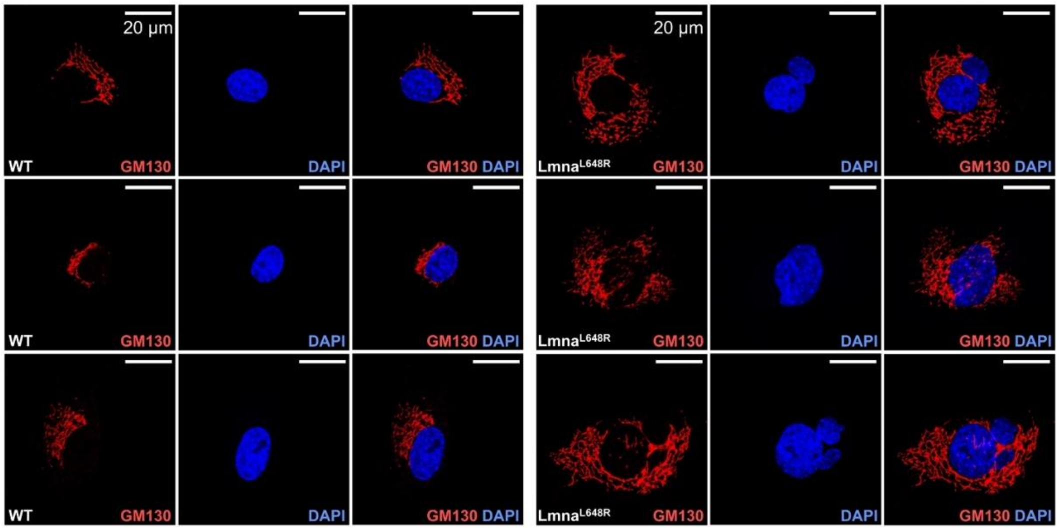

Supplemental Figure 13. ***Lmna^{L648R}*** **mutation causes Golgi dispersal.** Immunostaining of GM130, analyzing the Golgi of WT and *Lmna^{L648R}* calvarial cells. Scale bars, 20 µm.

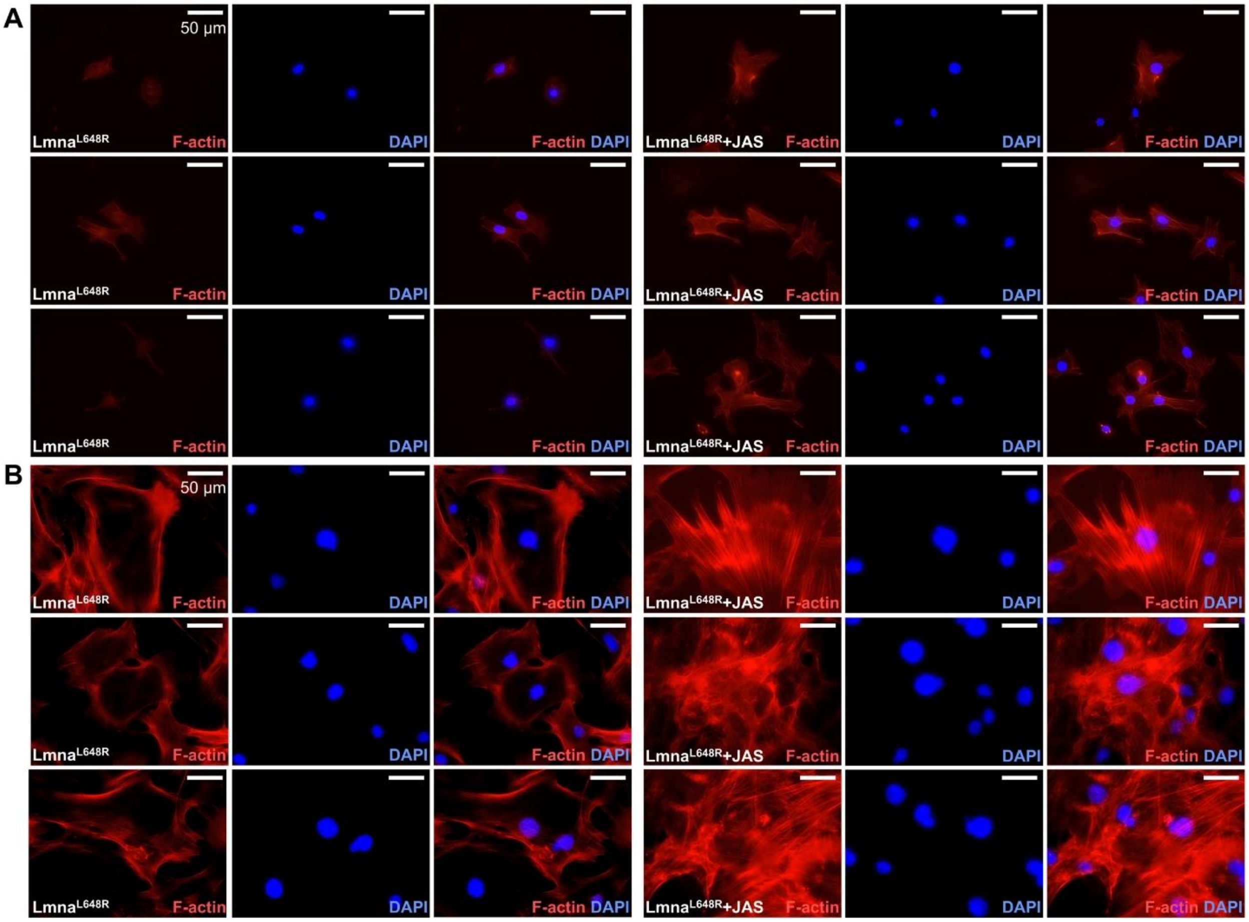

Supplemental Figure 14. JAS promotes actin polymerization in *Lmna^{L648R}* osteogenic cells. Filamentous actin (F-actin) staining analysis of the cytoskeleton in suture **(A)** and calvarial **(B)** cells isolated from *Lmna^{L648R}* mice, without or with the addition of JAS. Scale bars, 50 μm.

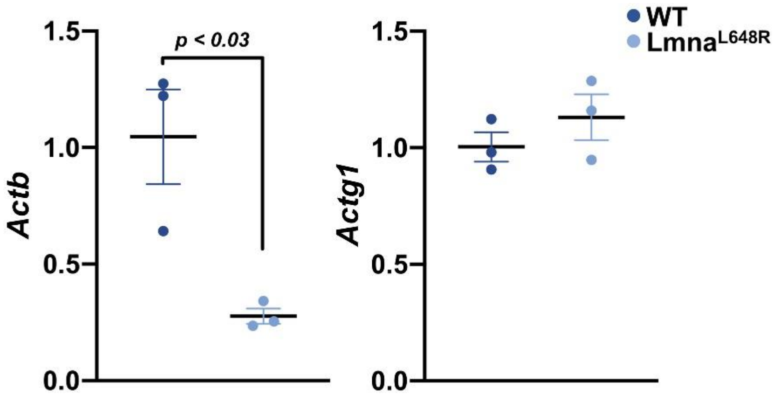

Supplemental Figure 15. The effect of JAS on actin gene expression in *Lmna^{L648R}* osteogenic cells. Plots show qRT-PCR results examining the expression of *Actb* and *Actg1* after culture of WT and *Lmna^{L648R}* mutant cells in differentiation media for 12 days (p-value as indicated, n = 3, mean ± SEM, two-sided Student's t-test).
